# An Intelligent Fusion Model with Portfolio Selection and Machine Learning for Stock Market Prediction

**DOI:** 10.1155/2022/7588303

**Published:** 2022-06-23

**Authors:** Dushmanta Kumar Padhi, Neelamadhab Padhy, Akash Kumar Bhoi, Jana Shafi, Seid Hassen Yesuf

**Affiliations:** ^1^School of Engineering and Technology, Department of Computer Science and Engineering, GIET University, Gunupur, India; ^2^KIET Group of Institutions, Delhi NCR, Ghaziabad 201206, India; ^3^Directorate of Research, Sikkim Manipal University, Gangtok 737102, Sikkim, India; ^4^AB-Tech eResearch (ABTeR), Sambalpur, Burla 768018, India; ^5^Department of Computer Science, College of Arts and Science, Prince Sattam Bin Abdulaziz University, Wadi Ad-Dawasir 11991, Saudi Arabia; ^6^Department of Computer Science, College of Informatics, University of Gondar, Maraki 196, Gondar, Ethiopia

## Abstract

Developing reliable equity market models allows investors to make more informed decisions. A trading model can reduce the risks associated with investment and allow traders to choose the best-paying stocks. However, stock market analysis is complicated with batch processing techniques since stock prices are highly correlated. In recent years, advances in machine learning have given us a lot of chances to use forecasting theory and risk optimization together. The study postulates a unique two-stage framework. First, the mean-variance approach is utilized to select probable stocks (portfolio construction), thereby minimizing investment risk. Second, we present an online machine learning technique, a combination of “perceptron” and “passive-aggressive algorithm,” to predict future stock price movements for the upcoming period. We have calculated the classification reports, AUC score, accuracy, and Hamming loss for the proposed framework in the real-world datasets of 20 health sector indices for four different geographical reasons for the performance evaluation. Lastly, we conduct a numerical comparison of our method's outcomes to those generated via conventional solutions by previous studies. Our aftermath reveals that learning-based ensemble strategies with portfolio selection are effective in comparison.

## 1. Introduction

Before the end of the twentieth century, low-frequency financial data were available for analysing and forecasting the stock market. Fewer professionals and academicians use these low-frequency data for their empirical studies, but as there are no sufficient related data available, the empirical research will not succeed [1]. Due to the rapid development of science and technology, the cost of data capture and storage has been reduced dramatically, which makes it easy to record each day's trading data related to the financial market. As a result, significant financial analysis of data has become a prominent area of research in economics and a variety of other disciplines [2].

With the recent rapid economic expansion, the quantity of financial activities has expanded, and their fluctuating trend has also become more complex. Asset prices trend forecast is a classic and fascinating issue that has piqued the interest of numerous academics from several fields. Academic and financial research subjects to understand stock market patterns and anticipate their growth and changes. Portfolio construction through competent stock selection has long been a critical endeavour for investors and fund managers. Portfolio enhancement and optimization have emerged among the most pressing issues in modern financial studies and investment decision-making in this era [3]. Portfolio development success is highly contingent on the future performance of financial markets. Forecasts that are realistic and exact can provide substantial investment returns while mitigating risk [4]. The prevailing economic and financial theory is the efficient market hypothesis (EMH) [5]. According to this hypothesis, forecasting the valuation of capital assets is challenging. However, according to past research, equity markets and yields can be predicted [6]. Before the invention of efficient machine learning algorithms, academics generated prediction models for research using a variety of alternative and econometric approaches [7]. Traditional statistical and econometric tools require linear models and cannot anticipate or analyse financial goods until nonlinear models are turned into linear models. Many studies have proven that nonlinearities arise in financial markets and that statistical models cannot effectively control them. With the rapid rise of AI and machine learning over the last decade, an increasing number of financial professionals have begun to analyse the index value of gaugeable models, have different requirements, and experiment with diverse methodologies [8]. K-NNs [9], Bayes classifiers [10], decision trees [11], and SVMs [12] are presently widely used for classification tasks [13]. However, in practice, these solutions fail to function when data are collected over an extended period, and storage space is limited (processing data at once are impractical).

Due to the ever-growing volume of incoming data, such as stock market indices, sensor readings, and live coverage, online learning has become highly significant [14]. When it comes to online learning, a system should absorb more training data without having to retrain from the beginning. Traditional AI frameworks, such as supervised learning tasks, usually work in a batch learning mode. A training dataset is supplied beforehand to train the model to use some learning algorithm. Due to the high cost of training, this paradigm necessitates the accessibility of the full training set before the learning assignment, so the learning process is frequently conducted offline [15]. In addition to being inefficient and low in both time and space costs, batch learning approaches have the disadvantage of being unable to scale for a range of applications since models would frequently need to be retrained from scratch for new learning data. With incremental learning, a learner attempts to acquire and improve the best predictor for a model as they go along through a sequential flow of information rather than using batch classifiers. Online learning overcomes the limitations of batch learning by allowing prediction models to be updated quickly in response to current data examples. Since machine learning jobs in real-world information analytic platforms tend to involve large volumes of information arriving at high speeds, online learning techniques offer a much more flexible and effective method for handling massive data inputs. In the real world, online learning can solve problems in several different application areas, just as traditional (batch) machine learning can.

Asset prices and economic forecasts are among the most complex and challenging activities in finance. Most traders depend on technical, fundamental, and quantitative analysis for making forecasts or creating price signals. With the advent of AI in different fields, its rippling effect may also be seen in finance and price forecasting. As stock prices are updated every second, there is always a possibility of a drift in the data distribution and rendering [16]. Continual advances in computational science and data innovation are essential to the globalization of the economy [17]. While numerous methods exist for estimating the cost of financial exchange, the latter has been the focus of the investigation. We may discover many issues and constraints in coming to more critical data even though there are many methods. Because classic analytical approaches have apparent flaws in dealing with nonlinear difficulties, several machine learning algorithms are being used in stock exchange inquiries [18].

Financial backers can make sound decisions, increase productivity, and reduce possible losses using a model capable of forecasting the growth path of a stock's value. As a result, accurate forecasting and stock market research have become more complex and less favourable. We must constantly improve our deciding approaches for stock price prediction. Previously, several domestic and international researchers were dedicated to developing measurable monetary frameworks to forecast index growth. Before the advent of expert AI computations, analysts routinely used a variety of statistical approaches to create expectation models. A stock market prediction can be made with linear and nonlinear models. Most linear models are based on statistics, while most nonlinear models are based on machine learning techniques [19]. In principle, customary monetary structures and the arising automated thinking model might accomplish stock expense estimating, but the expectation sway is very astounding [20]. Observing systems with work on predicting future outcomes through model combination and inspection is advantageous for some analysts, and it also has excellent speculative value [21]. In reality, authentic data may be coordinated into monetary systems to forecast future data. For instance, assuming the stock worth check is more prominent, the model predicts that the future share price will be higher than the end-of-day price will climb. Monetary allies might choose to stay retaining the shares to obtain a higher return on investment [22]. If the asset value guesstimate is less than the day's end price, the share price will likely fall later. As a result, developing a monetary framework to recognize asset value measuring is very feasible [23]. Furthermore, if you can correctly forecast the asset price movements and price flow patterns, it has a significant incentive for governments, listed companies, and private financial backers [24].

Recently, there has been a growth in research that looks at the path or pattern of financial market changes. Presently, the examination is advancing by inspecting the interest and pattern of securities exchanges. Academics have long been interested in equity market forecasting as an appealing and challenging subject. The amount of information that is available daily continuously increases; as a result, we are confronted with new issues in handling data to extract information and estimate the impact on asset values. There is always a challenge and disagreement when determining the optimal strategy to forecast the stock market's daily return trend. As the study aims to anticipate the future market, this study topic has a self-sabotaging behaviour that has proven to be fascinating and prevalent for stock market forecasting. Researchers can always discover industry secrets and analyse the market using their unique methods, thanks to the fast development of machine learning models, techniques, and technologies. The machine learning models can improve their prediction performance by identifying suitable feature selections. A poor feature selection reduces the model's performance and results in biased outcomes. Developing a reliable forecasting technique capable of identifying risk factors and providing favourable and unfavourable market direction is as important as appropriate feature extraction throughout the modelling procedure.

The purpose of this study was to develop an online learning framework based on machine learning that can reduce investment risk (by constructing an optimal portfolio) and make a predictive judgement regarding the direction of the selected indices. In addition, this study provides a new method for minimizing investment risk by building a framework that combines the mean-variance model for selecting stocks with minimal risk and an online learning framework for index forecasting.

This framework, in particular, has two major phases: portfolio selection and stock prediction. This study's primary contributions are summarized as follows:

- Compared with previous studies on portfolio development and machine learning-based forecasting strategies in general, it is always a top task to find the hidden features. So, the suggested approach has some unique combination of features generated from the raw transaction data with less effort on the human being.
- The system is intended for real-world use. Therefore, we adopted a unique framework that combines the mean-variance model for portfolio development and the online learning model for financial market prediction.
- Our experiment examined the performance of four different geographical reasons' health sector equities throughout volatility stress and smooth trending periods, as well as the durability of financial crises and clustering. For this objective, a large amount of data was collected over a lengthy period.

The remainder of the article can be deduced from the information provided below.  Section 2 portrays the related work, Section 3 depicts the materials and techniques, and Section 4 investigates our proposed framework.  Section 5 focuses on exploratory research findings and discusses the most significant discoveries made during our investigation. Last but not least, in Section 6, we discuss the conclusion section of our work and the future scope of our research.

## 2. Related Work

Investing proponents and professionals have long held that stock price movements are unpredictable. The phrase “efficient market hypothesis” was coined by Fama [25] and gave rise to this point of view (EMH). The nonstationary and dynamic nature of financial market data, according to Fama, makes it impossible to make predictions about the capital market [25]. According to the EMH, the market reacts immediately to new information about financial assets. As a result, it is impossible to break into the market. According to Shiller [26], the financial sector entered the 1990s when academics dominated behavioural finance. From 1989 to 2000, Shiller's [26] study found that fluctuations in the stock market were driven by investor mood. When Thaler [27] predicted that the Internet stock boom would collapse, he criticized the generally accepted EMH of accepting all financial supporters as usual and making plausible forecasts about the future.

On the other hand, behavioural finance argues that stock market movements are always based on real knowledge, according to Shiller [26]. Shiller [26] showed that short-term stock prices are unpredictable, while long-term stock market movements are predictable. Fundamental and technical variables are both important when it comes to financial market forecasting [28, 29]. The entire analysis considers how much money the company has left, how many workers there are, how the directorate makes decisions, and what the company's yearly report looks like [30]. It also takes into account things such as unnatural or catastrophic events, as well as information about politics. People look at the main things are the company's GDP, CPI, and *P*/*E* ratios [31]. Stock market forecasting can benefit from a fundamental strategy that prioritizes the long term above the immediate [32]. Specialized observers [33] use trend lines and technical indicators to forecast the securities market for specialized observers [33]. Technical analysts can make educated guesses using mathematical algorithms and previous price data [34].

Researchers now have more resources to work with as AI techniques improve and datasets become more widely available, opening up new directions for investigation. According to Marwala and Hurwitz [35], advances in AI technologies have influenced the EMH and fuelled a need to learn from the market. According to a growing corpus of studies, capital markets can be predicted to some extent, according to a growing corpus of studies [36, 37]. Consequently, investors have the chance to minimize their losses while maximizing their earnings while dealing with the stock market [38]. Recent research suggests that statistical and machine learning are two distinct approaches [39].

Statistical techniques were utilized before machine learning to analyse market trends and analyse and forecast stocks. To assess the financial market, several statistical models are employed [40–42]. Traditional statistical approaches have struggled tremendously, and machine learning approaches are beginning to develop to circumvent the drawbacks of conventional statistical methods [43]. Numerous machine learning algorithms have been used to anticipate the stock market [44–49]. Prior research has established that machine learning techniques outperform all other predicting stock market directionality [50]. Traditional models are less flexible than AI approaches [51, 52]. Several machine learning algorithms have been investigated in the past [53, 54]. Some examples are logistic regression, SVM, K-NN, random forests, decision trees [34, 37, 40], and neural networks [37, 38]. As described in the literature, SVM and ANN are the most frequently used algorithms for stock market forecasting. A long-term financial market forecasting classification system was proposed by Milosevic et al. [55]. They say that a stock is excellent if its value improves by 10% in a fiscal year; otherwise, it is bad. Eleven fundamental ratios were recovered throughout the model-building process and are used as input features by several algorithms. They found that the random forest had an *F* score of 0.751 in differentiation using naive Bayes and SVM. Choudhury and Sen [56] trained a back propagation neural network and a multilayer feedforward network to forecast the stock value. A regression value of 0.996 was obtained using their proposed model.

Boonpeng and Jeatrakul [57] developed a multi-class categorization problem to determine whether a stock is a good investment. According to their findings, one-against-all neural networks beat the traditional one-against-one and classic neural network models with a 72.50% accuracy rate. According to Yang et al. [58], an effective forecasting model requires understanding the nonlinear components of stock. Multiple machine learning models for stock market direction prediction have been created, as Ballings et al. [59] noted. In addition to datasets from European businesses, they used a range of ensemble machine learning algorithms. They also used neural networks and logistic regression. Finally, using the random forest approach, they could predict the long-term fluctuations in the stock market using their dataset. According to Leung et al. [60–62], accurate forecasts of the growth of the stock worth list are essential for the creation of effective trading approaches such as financial backers that can protect against the predicted dangers of the securities exchange. Even if only a little accuracy is gained, anticipating execution is a considerable benefit. When it comes to predicting the financial markets, machine learning techniques often fall into one of two camps: predicting the stock market using solitary machine learning algorithms or employing many models. According to a number of studies, ensemble models are more accurate than solitary forecasting models. Only a few studies have looked into ensemble models [63].

Many ensemble approaches have been developed in machine learning platforms to improve predicting performance and decrease bias and variance trade-offs [64]. The most often used algorithms for machine learning-based ensemble learning include AdaBoost [65], XGBoost [66], and GBDT [67]. In Nobre and Neves [66], an XGBoost-based binary classifier is introduced. The results demonstrate that the framework may provide greater average returns. Furthermore, a stock forecasting model employing technical indicators as input features was proposed by Yun et al. [68]. According to the researchers, their XGBoost models outperformed both SVM and the ANN. Based on risk categorization, Basak et al. [25] created a methodology for forecasting whether the stock price will rise or fall. When employing random forest and XGBoost classifiers, researchers demonstrated that hybrid models perform much better with the right set of indicators as input features for a classifier. Ecer et al. [63] claim that ensemble machine learning approaches are superior to individual machine learning models in terms of performance. Multilayer perceptron, genetic algorithms, and particle swarm optimization are included in two new methods proposed by Ecer et al. [63]. A total of nine technical indicators were used to train their model and resulted in RMSEs of 0.732583 for MLP–PSO and 0.733063 for MLP–GA, respectively. According to the researchers, a combination of machine learning techniques can improve prediction accuracy.

Yang et al. [69] presented a feedforward network composed of many layers for Chinese stock market forecasting. Back propagation and Adam algorithms were used to train the model, and an ensemble was created using the bagging approach. The model's performance may be enhanced by further normalizing the dataset. Wang et al. [70] constructed a combined approach that forecasts the financial markets every week using BPNN, ARIMA, and ESM. In predicting stock market direction, they found that hybrid models beat regular individual models with an accuracy of 70.16 percent. Finally, Chenglin and colleagues [71] proposed a model for forecasting the direction of the fiscal market. According to the researchers, mixed models, which included SVM and ARIMA, outperformed standalone models. Tiwari et al. [72] proposed a hybrid model that combines the Markov model and a decision tree to forecast the BSE, India, with an accuracy of 92.1 percent. Prasad et al. [39] investigated three algorithms, XGBoost, Kalman filters, and ARIMA, as well as two datasets, the NSE and NYSE. First, they looked at how well individual algorithms could predict and how well a hybrid model they made with Kalman filters and XGBoost worked. Finally, they looked at four models and found that the ARIMA and XGBoost models did well on both datasets, but the accuracy of the Kalman filter was not consistent. Jiayu et al. [62] developed a combined LSTM and attention mechanism, called WLSTM + attention, and demonstrated that the suggested model's MSE became less than 0.05 on three independent measures. Moreover, they asserted that proper feature selection might enhance the model's forecasting accuracy.

Portfolio enhancement is the circulation of abundance among different assets, wherein two parameters, in particular, anticipated returns and risks, are vital. The ultimate goal of financial backers is usually to increase the returns and decrease the risks. Usually, as the return margin increases correspondently, the risk margin also increases. The model introduced by Markowitz [73] is popularly known as the mean-variance (MV) model, whose main objective is to solve the problems during portfolio optimization. The main parameter of this model is means and variances quantified by returns and risks, respectively, which facilitates financial supporters to strike a balance between maximizing expected return and reducing risk. After the exploration of Markowitz's mean-variance model, some researchers tried to develop a modified version of this model in different ways: (i) an optimized portfolio selection with respect to multi-period [74–76] and (ii) introducing alternate risk assessment methods. The safety-first model [77], the mean-semi-variance model [78], the mean absolute deviation model [79], and the mean-semi-absolute deviation model [80] are all examples. (iii) Many real-world constraints, such as cardinality constraints and transaction costs, were also included in the study. [81–84]. Nonetheless, the above examinations focus harder on the improvement and extension of the mean-variance model; however, they never consider that it is essential to select high-quality assets for creating an optimal portfolio. The investment strategy process generally said that if we provide high-quality assets as input, there is a quiet assurance that we construct a reliable optimized portfolio. In the last few years, a few studies have been done to ensure that the asset selection and the portfolio determination models work together.

For an investment decision, Paiva et al. [85] develop a model. First, they use an SVM algorithm to classify assets, and then, they use the mean-variance model to make a portfolio. A hybrid model proposed by Wang et al. [86] is a combination of LSTM and Markowitz's mean-variance model for optimized model creation and asset price prediction. These investigations showed that the mix of stock forecast and portfolio determination might give another viewpoint to financial analysis. So, in our current study, we use the mean-variance model for portfolio selection and determine individual assets' contribution towards our model-building process.

Machine learning strategies have been broadly utilized for classification-related issues [13]. A couple of techniques and models are discussed in the above literature. However, instead of dealing with theoretical concepts if we deal and work with the practical environment, in the stock market the data are coming in continuously over a long duration time and the execution of current data at once each time is impossible, so these techniques are not working properly for forecasting in a real environment. As a result, online learning is becoming increasingly important in dealing with never-ending incoming data streams such as sensor data, video streaming data, and financial market indexes [14]. So, when it comes to online learning, a system should absorb extra training data without retraining from the start.

During the online learning process, the continuous data flows are coming in a sequence, and the predictive model generates a prediction level on each round of data flows. Then, according to the current data, the predictive online learning model may update the forecasting mechanism. Perceptron [87] is a basic yet effective incremental learning algorithm that has been widely researched to improve its generalization capability. Crammer et al. [88] introduced the passive-aggressive (PA) algorithm, which is faster than perceptron and sometimes shows more promise than perception. When the new sample comes in, it changes the model to ensure it does not lose too much data and that it is almost the same as the old one. The retrained model guarantees that it has a minimal loss on the current sample and is similar to the present one. A few online machine learning approaches have been developed to cope with massive streaming data. New rules may be discovered when new data arrive, while current ones may be revised or partially deleted [89]. In the training phase of traditional machine learning algorithms, each sample was considered equally valuable. However, in real-world applications, different samples should contribute to the decision boundary of participating classifiers in distinct ways [90]. Perceptron-based projection algorithm was proposed by Orabona et al. [91]; however, the number of online hypotheses is limited in this technique by projecting the data into the space encompassed by the primary online hypothesis rather than rejecting them. In ALMAp [92], the maximum margin hyperplane is estimated for a collection of linearly separable data.

Furthermore, SVMs have been updated for numerous iterative versions [93–95], which define a broad online optimization issue. For example, Laughter [96] introduced two families of image classification online developing classifiers. The created classifiers are first given training upon specific pre-labeled training data before being updated on newly recorded samples. In [97], a robust membership computation approach that works extraordinarily when confronted with noisy data was given. However, many membership generation algorithms are designed for specific data distributions or presuppose batch delivery of training samples. Because the early phases of distribution information are erroneous, transferring such approaches directly to online learning may provide additional issues. More significantly, when a fresh instance is obtained, the new decision boundary must be computed using the complete existing training set, which takes more time. As far as we know, very few articles have been written about the subject. As a result, a solid and efficient framework of incremental forecasted model based on the stock market is required for online classification. Overall, this research line has demonstrated significant promise for incremental model parameter modification and excellent understand ability of online learning systems in dynamically changing contexts.  Table 1 shows the numerous research studies conducted based on batch learning techniques.

When we forecast the financial market using these methodologies, the literature mentioned above has some shortfalls, like these techniques follow the traditional batch learning techniques, which further can be improved by the help of online learning techniques. Moreover, some literature faces imbalanced classification problems when multiple indices are examined from different countries' indices. Therefore, we selected quality-based stocks using the mean-variance model instead of focusing on the randomly selected stock for the experiment. So, we have introduced a framework that can handle the situations outlined above.

## 3. Materials and Methods

Before discussing our framework, we have emphasized the significant methodologies and datasets that will be employed in our proposed framework. Online learning or incremental learning is a machine learning approach for sequential data in which the learner attempts to develop and demonstrate the best predictor for each new dataset. Allowing the prediction model to be modified quickly for any current data instances, online learning makes up for the weaknesses of batch learning. As we all know, stock market data always come into existence sequentially and regularly. As a result, the batch learning process suffered greatly. So, we constructed two online learning algorithms for our experimental goals, which are briefly described below.

### 3.1. Perceptron

The perceptron algorithm is the most ancient method for online learning. The perceptron algorithm for online binary classification is described in Algorithm 1.

In general, if a specific margin can separate the data, the perceptron technique should result in a maximum of (*R*/*yp*)^2^ errors, where the margin *λ* is specified as *λ*=min_*td*∈[*TD*]_|*xi*_*td*.._*wg*^*∗*^| and *R* is a constant such that ∀_*td*_ ∈ [*TD*], *xi*_*td*_ ≤ *R*. The higher the margin *λ*, the narrower the error bound.

Numerous variations of perceptron algorithms have been presented in the literature. A straightforward modification is the normalized perceptron method, which varies only in its updating rule:

(1) $$\begin{array}{c} wg_{td+1}=wg_{td}+yp_{td}\frac{xi_{td}}{||xi_{td}||}. \end{array}$$

### 3.2. Passive-Aggressive Classifier

When a new piece of data comes in, the model is updated to ensure that the new piece of data does not get lost and that the model is close to the one already there [15, 105, 106].

This algorithm falls under the family of first-order online learning algorithms, and it works with the principle of margin-based learning [95].

Given an instance **xi**_**tr**_ at round tr, the passive-aggressive generates the optimization as follows:

(2) $$\begin{array}{c} WE_{tr+1}=\text{arug}\overset{\underset{\;}{w\in R^{d}}}{\min i}\frac{1}{2}||WE-WE{}_{tr}||{}^{2},\\ \text{s}\ell_{1}\left(WE\right)=0, \end{array}$$

where *ℓ*_1_(*WE*)=max*i*(0,1 − *yp*_*tr*_*WE*.*xi*_*tr*_) is the hinge loss of the classifier. When the hinge loss is zero, i.e., *WX*_*tr*+1_=*WX*_*tr*_when*ℓ*=0, then the classifier is passive and the classifier is treated as aggressive, and when loss is nonzero, then the algorithm is named as “passive-aggressive” (PA) [95]. So, the aim of the passive-aggressive classifier is to update the classifier *WX*_*tr*+1_ and stay close to the previous one.

In particular, PA aims to keep the updated classifier *WX*_*tr*+1_ stay close to the previous classifier (“passiveness”) and make sure all incoming instances are correctly classified by updating the classifier.

It is critical to recognize a significant distinction between PA and perceptron algorithms. Perceptron updates only when a classification error occurs. However, a PA algorithm updates aggressively anytime the loss is nonzero (even if the classification is correct). Although PA algorithms have equivalent error limitations to perceptron algorithms in principle [95], they frequently outperform perceptron considerably practically.

### 3.3. Modern Portfolio Theory

Modern portfolio theory (MPT), sometimes referred to as mean-variance analysis, is a mathematical framework for designing an asset portfolio to maximize expected returns for a given level of risk. It is a formalization and extension of diversification in investing, which maintains that possessing a diverse portfolio of financial assets is less risky than owning only one type. Its fundamental premise is that an asset's risk and return should not be evaluated in isolation but rather in connection to contributing to the portfolio's overall risk and return. Asset price volatility is used as a proxy for risk. Economist Harry Markowitz [73] popularized MPT in a 1952 article for which he was later given the Nobel Memorial Prize in Economic Sciences, which became known as the Markowitz Prize.

As per the following multi-objective optimization formula, the mean-variance model maximizes profits and reduces risks simultaneously.

(3) $$\begin{array}{c} \min\;\overset{N}{\underset{k-1}{\sum }}\overset{N}{\underset{l-1}{\sum }}z_{k}z_{l}\sigma_{kl},\\ \max\;\overset{n}{\underset{k=1}{\sum }}z_{k}\mu_{k},\\ \text{s}\left\{\begin{array}{c} \overset{N}{\underset{k=j}{\sum }}z_{1},\\ 0\leq z_{k}\leq 1,\forall k=1.....N, \end{array}\right. \end{array}$$

where *σ*_*kl*_  denotes the correlation between assets *k* and *l*, *z*_*k*_*andz*_*l*_ denote the fraction of the original value; and *μ*_1_ is the expected return on asset *k*.

### 3.4. Imbalanced Data Handling Techniques

Imbalanced data distribution is frequently used in machine learning and data science. It occurs when the number of observations in one class is considerably more or smaller than the number of observations in other classes. However, because machine learning algorithms maximize accuracy by decreasing mistakes [107], they ignore class distribution. In more technical words, if our dataset includes an unequal data distribution, our model is more susceptible to situations in which the minority class has very little or no recall.

#### 3.4.1. Synthetic Minority Oversampling Technique (SMOTE)

You can use synthetic minority oversampling technique (SMOTE) to deal with not evenly split up data. It tries to even things out by adding minority class examples at random through replication.

*Step 1.* By calculating Euclidean distances between *x* and each other sample in set *A*, we define the minority classes set *A* for each *k*-nearest neighbors of *x*.
*Step 2.* To determine the sampling rate *N*, an imbalanced proportion is calculated. The set A1 is constructed by randomly selecting *N* (i.e., *x*1, *x*2,…, *xn*) from its *k* closest neighbors, for each *x* ∈ *A*.
*Step 3.* To make a new example for each complex *x*_*k*_ ∈ *A*_1_, (*k*=1,2,3....*N*), the following formula is used:
  (4) $$\begin{array}{c} x^{\prime }=x+\text{rand}\left(0,1\right)*\left|x-x_{k}\right|, \end{array}$$
   where rand (0, 1) denotes a random value between 0 and 1.

SMOTE is a well-known data preparation approach for addressing the issue of class imbalance. Sun et al. [103] provide the SMOTE technique, which generates new samples by identifying the *k*-nearest neighbors of each minority class sample and randomly interpolating between them to achieve sample class balance before training classifiers [103]. SMOTE, one of the most often used oversampling techniques for dealing with class imbalance, provides new minority class samples to balance the training dataset, hence enhancing the model's classification performance. SMOTE creates extra minority class samples combined with the initial training set to form an equitable training set. As an illustration of how SMOTE may be used to address a class imbalance in our prediction model, the SMOTE approach can be used to batch balance the original training dataset before starting the ensemble approach.

### 3.5. Evaluation Matrices

To assess the efficiency of the suggested model, we used performance matrices such as accuracy, receiver operating characteristic (ROC) curve (AUC), and F score. As a result, we evaluated the framework's performance using a mixture of matrices rather than a single one. The following are the performance matrices.

(5) $$\begin{array}{c} \text{precision}=\frac{tr_{p}}{tr_{p}+fa_{p}},\\ \text{recall}=\;\frac{tr_{p}}{tr_{p}+fa_{n}},\\ \text{ F score}=2\frac{\text{precision}*\text{recall}}{\text{precision}+\text{recall}},\\ \text{accuracy}=\frac{tr_{p}+t_{rn}}{tr_{p}+tr_{n}+fa_{p}+fa_{n}}, \end{array}$$

where *tr*_*p*_ denotes the total number of real positive values; *tr*_*n*_ denotes the total number of real negative values; *fa*_*p*_ denotes the total number of false-positives values; and *fa*_*n*_ denotes the total number of false-negative values.

According to Shen and Shafiq [101], the area under curve is an acceptable assessment matrix for classification issues; as the AUC value grows, so does the model's prediction ability.

#### 3.5.1. Hamming Loss

In general, there is no magical metric that is the best for every problem. In every problem, you have different needs, and you should optimize for them. The Hamming loss is the proportion of wrong labels compared with the total number of labels. Hamming loss is calculated as the Hamming distance between actual and predicted values. Generally, the Hamming loss is related to imbalance classification problems.

### 3.6. Instrumentation and Systems Employed

We used the Python open-source environment and Google Colab for our scientific experiment purposes for our suggested framework. Here, we used the Scikit library to access the predefined library functions related to machine learning models and the TA-Lib library to find the technical indicators used in our experiment. The complete development procedure was run on an Intel CPU (Core-i5-1035G1, 1.19 GHz) processor, with RAM installed of 8 GB, and the OS used 64 bit Windows.

### 3.7. Dataset

In particular, for our innovations, five different health sector indexes have been selected from four different stock markets of four different nations, namely London, Germany, France, and America. The data on equity indexes are updated daily. In addition, each trading day's equity indexes are included in all datasets. Initially, we chose five high-quality companies from each nation based on their performance over time and asset size. When we are considering the stock market for forecasting, as we know, the nature of the dataset is random. So, if we take only one dataset for our experiment, we may not conclude that our model can also perform better on other datasets. So, for making a model as a general one we will suggest multiple datasets. The details of the stock are shown in Tables 2 and 3.

## 4. Proposed Framework

**Figure 1:**
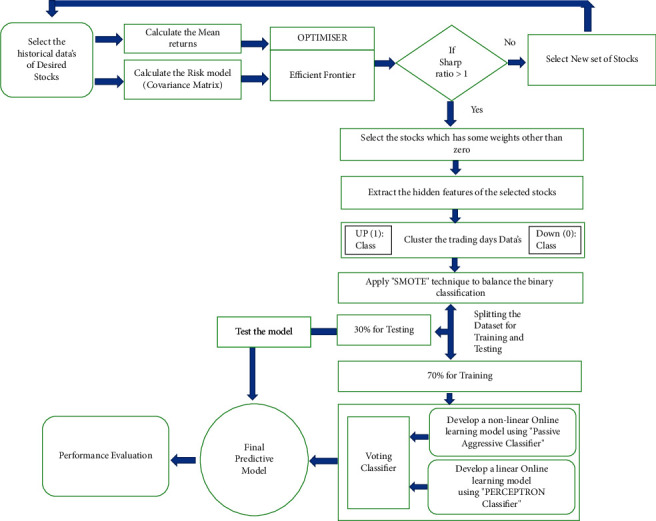
Proposed framework.

This study suggests a new technique to minimize the investment risk by developing a framework that is a combination of the mean-variance model for selecting minimal risk-based stock and a machine learning-based online learning model for stock forecasting. The proposed framework is shown in Figure 1. This framework, in particular, has two major phases: portfolio selection and stock prediction. So, during our framework setup, our empirical research was gone through four stages:

1. Initial asset selection.
2. Developing a mean-variance model for return prediction and final stock selection for an experiment.
3. Predictive model setup.
4. *Outcome Evaluation*. The Python programming language is used to prepare the computations, Scikit-learn is used to configure and train the online predictive model, and PyPortfolioOpt is used to implement the optimization strategies for finding the most valuable stocks for investment.

Initially, we selected 20 stocks for four different geographic reasons; they are enumerated in Table 2. The stock selection is based on the previous and current performances according to the market capitalization, and those stocks have been selected for their market existence for more than 20 years.

After selecting individual 20 stocks for different geographic reasons, we have to find the potential stocks that give a minimum loss and a maximum profit for an investor. Finally, those will be taken for the final experiment. For the selection of potential stocks, we examine the current portfolio theory, sometimes referred to as the mean-variance model, which was presented by Markowitz [73].

From the selected stocks' historical datasets, we have collected for one year. We have extracted the mean return and covariance matrix of each stock according to geographic reasons from the historical price. Then, the extracted parameters, i.e., mean return and covariance matrix, are passed to the mean-variance model using the efficient Frontier optimizer. After implementing this optimization technique, we got the following potential stocks for future productivity: here, the following Tables 4–7 show the weights of each stock according to the geographic reason.

According to the modern portfolio theory, stocks with weight values other than zero are considered potential stocks. So, from the Tables 4–7, we have recorded the weights of each stock, and finally, we have to select only stocks that have weights greater than zero. To minimize the risk factor again, we have added one condition that those group stocks with a sharp ratio of more than 1 will be a great advantage for investors in choosing whether or not to purchase or sell a particular stock. Now, it is time to extract the real stocks, which will be carried out for our experiment.

After successfully selecting potential stocks, our next objective is to create an online training approach before developing an online training approach. It is crucial to find the essential features for finding the hidden truth behind our stock. Data preprocessing makes the dataset more powerful and makes sense for a machine learning model. Here, we are highlighting the steps.

### 4.1. Data Preprocessing

An earlier study of related topics lacked explicit instructions for picking relevant input features to predict the index's flow direction. As a result, we can confidently assert a hidden behaviour behind every technical feature. For example, according to Weng et al. [18], investors use covert behaviour to analyse the present circumstances and determine whether to purchase or sell. Ultimately, given their opinion on the indicators that evaluate the concealed performance of these input data, it is possible to anticipate the fiscal market uniquely. As a result, we used indicators in our investigation and other elements to forecast asset price movement.

The raw dataset must be preprocessed once it has been received. As part of the data preprocessing, we followed the following steps:

(1) In general, the index collected from the online site has specific preexisting attributes such as open, close, low, high, and soon. Therefore, we needed to deal with the null and missing values with the dataset in hand.
(2) We extracted eight technical indicators from the previously stated line in the second phase. In addition, our process included two additional features: the variation between the opening and closing prices of a stock on a given day, which represents both a growth and a fall in its value, as well as the difference between the high and low price, which represents the volatility of that day's stock price.
(3) As part of our response anticipated variable, we created a binary feedback variable for individual trading days, i.e., *Dn* 0 and 1. The forecasted feedback variable on the *N*th day is computed as follows:
    If Op_*n*_ < Cl_*n*_
    Then
     *D*_n_ = 1
    Else
     *D*_n_ = 0
    End If

In this case, *D*_*n*_ is our forecasted variable since we used the prediction label “TREND.” On the *n*th day of the index's life, Op_*n*_ is its opening price and Cl_*n*_ is its closing price. For instance, if the *D*_*n*_ returns a value of “1,” then the fund's value will rise, but if the *D*_*n*_ returns a value of “0,” then the fund's value will fall. Traders and researchers use the (Ta-Lib) library to calculate technical indicators in the technical analysis [108]. We use the VIF technique to find the best set of features out of many.

### 4.2. Extracted Features

#### 4.2.1. SAR Indicator

An indicator called the parabolic SAR is a way to see how the price of a specific thing will change over time.

#### 4.2.2. Parabolic SAR Extended

It is an indicator designed for opsonists as it is reactive at the beginning of the trend but then remains little influenced by the movements. Although significant, that does not change the current trend. Buy signals are generated when the indicator is above 0; sell signals are generated when the indicator is below 0.

#### 4.2.3. Aroon Indicator

The Aroon indicator determines whether a price is trending or trading inside a range. Additionally, it can show the start of a new trend its strength and assist in anticipating transitions from trading ranges to trends.

#### 4.2.4. The Balance of Power (BOP)

The balance of power (BOP) indicator measures a price trend by evaluating the strength of buy and sell signals, determining how strongly the price moves between extraordinarily high and low levels. The BOP oscillates between −1 and 1, with positive values indicating more substantial buying pressure and negative values indicating intense selling pressure. When the indicator gets closer to zero, it shows that the buyers' and sellers' strength is equating.

#### 4.2.5. The Directional Movement Index

The DMI is a useful metric that is used to cut down on the number of false signals. It analyses both the degree and direction of a price movement. The greater the spread between the two main lines, the more influential the trend.

#### 4.2.6. The Chaikin *A*/*D* Oscillator

The indicator examines the line of moving average convergence-divergence that shows how much money is added or taken away. A cross above the accumulation-distribution line means that people in the market are buying more shares, securities, or contracts, which is usually a good thing.

#### 4.2.7. OBV

On balance volume (OBV) is a straightforward indicator that uses turnover and pricing to determine how much people buy and sell. There is much-buying pressure when positive volume outnumbers negative volume, and the OBV line rises.

#### 4.2.8. True Range

It looks at the range of the day and any different from the previous day's close price.

#### 4.2.9. COS

Vector cosine calculates the trigonometric cosine of each element in the input array.

#### 4.2.10. Open

This is a feature that has been there for a long time. It shows the stock price at the start of every day.

#### 4.2.11. Open-Close

Under this instance, the disparity between the entry and exit prices is clearly shown in all of the transactions that happen each day.

#### 4.2.12. High-Low

This feature shows how volatile each trading day is. On that day, it is the gap between the top and low price points.

#### 4.2.13. Close

At the close of the stock each day, this feature shows the stock price. Again, this is a feature that has been there before.

#### 4.2.14. Volume

This feature shows each day's total buying and selling quantity on each trading day.

As it is a binary classification problem whether the stock goes up or down, we found different stocks from different geographic regions during the preprocessing data stage, which leads to a data imbalance problem. So to avoid this problem, we used the SMOTE technique to balance the dataset. Finally, as part of the data processing stage, we used the scaling approach to normalize the characteristics that would be fed to our model. After acquiring a balanced dataset, 75 percent of the dataset was used for training and 25 percent for testing; we divided it into these two groups. As we know, during ML model training there is a chance of overfitting, so technically, during our practical experiment, we adopted the cross-validation technique to avoid overfitting issues.

As far we discussed the demerits of offline learning models, we have implemented two online learning models that can allow the prediction model to be upgraded quickly for any current data instances. Consequently, online learning algorithms are substantially more efficient and scalable than traditional machine learning algorithms to be applied to a wide range of machine learning problems encountered in actual data analytic applications. So, we have used two online learning algorithms. One is a perception, and the other one is a passive-aggressive classifier. Instead of developing a single predictive model for forecasting, we combine the models' predictive capabilities and pass them to a voting classifier. Finally, the voting classifier merged the performances and built a highly reliable online predictive forecasting model.

## 5. Results and Discussion

To evaluate the suggested method's performance, we employed 10 potential indices for different geographic reasons, as reflected in Table 3. In this research, we assume that optimization techniques represented by the mean-variance model are well suited to enhancing the Sharpe ratio for building a portfolio of different geographically reason-based stocks. For selecting potential stocks, the primary performance measure that we use is the Sharpe ratio, and we calculate the weights of each index using the mean-variance optimization technique. The results are reflected in Tables 4–7. To test our online learning predictive model, we have selected different geographic stocks whose performance measures are given below according to the geographic reasons.

As shown in Table 8, our proposed model was performed on three American stock indices, likely UNH, NVO, and DHR, whose performances and accuracy measures are shown in Table 8. The recorded results found that the UNH index has a training accuracy of 99.16, while the testing accuracy is 99.60. The recorded AUC score is 99.6041, and the Hamming loss is 0.00399. The precision value recorded about group 0 is 0.99 and to group 1 is 1.00, whereas the recall value recorded pertaining to group 0 is 1.00 and to group 1 is 0.99. Finally, the *f*1 score related to group 0 is 1.00 and about group 1 is 1.00. The NVO index has a training accuracy of 99.68, whereas the testing accuracy is 99.73. The recorded AUC score is 99.7322, and the Hamming loss is 0.002368. The precision value recorded in group 0 is 0.99 and related to group 1 is 1.00, 0, whereas the recall value recorded in group 0 is 1.00 and related to group 1 is 0.99. Finally, the *f*1 score related to group 0 is 1.00 and about group 1 is 1.00. The DHR index has a training accuracy of 99.47, while the testing accuracy is 99.36. The recorded AUC score is 99.3724, and Hamming loss is 0.00635. The precision value recorded about group 0 is 0.99 and group 1 is 1.00. In contrast, the recall value recorded about group 0 is 1.00 and on group 1 is 0.99, and finally, the *f*1 score about group 0 is 99.00 and related to group 1 is 99.00.  Table 9 shows the confusion matrix.

Our proposed model was performed on four French stock indices, likely CVS.F, EWL.F, MTO.F, and TN8.F, whose performances and accuracy measures are shown in Table 10. From the recorded results, we found that the CVS.F index has a training accuracy of 99.72, whereas the testing accuracy is 99.49. The recorded AUC score is 99.50, and the Hamming loss is 0.00508. The precision value recorded pertaining to group 0 is 0.99 and to group 1 is 1.00, whereas the recall value recorded pertaining to group 0 is 1.00 and to group 1 is 0.99. Finally, the *f*1 score related to group 0 is 99.00, and pertaining to group 1 is 1.00. By looking at the table, the EWL. *F* index has a training accuracy of 99.87 percent, whereas the testing accuracy is 99.61 percent. The recorded AUC score is 99.60, and the Hamming loss is 0.00386. The precision value recorded pertaining to group 0 is 0.99 and pertaining to group 1 is 1.00. In contrast, the recall value recorded pertaining to group 0 is 1.00 and pertaining to group 1 is 0.99, and finally, the *f*1 score pertaining to group 0 is 1.00 and pertaining to group 1 is 1.00. The MTO.F index has a training accuracy of 99.94, whereas the testing accuracy is 99.91. The recorded AUC score is 99.91, and the Hamming loss is 0.00086. The precision value recorded pertaining to group 0 is 1.00 and pertaining to group 1 is 1.00, whereas the recall value recorded pertaining to group 0 is 1.00 and pertaining to group 1 is 1.00, and finally, the *f*1 score pertaining to group 0 is 1.00 and pertaining to group 1 is 1.00. The TN8.F index has a training accuracy of 99.81, whereas the testing accuracy is 99.84. The recorded AUC score is 99.84, and Hamming loss is 0.00151. The precision value recorded pertaining to group 0 is 1.00 and pertaining to group 1 is 1.00, whereas the recall value recorded pertaining to group 0 is 1.00 and pertaining to group 1 is 1.00. Finally, the f1 score pertaining to group 0 is 1.00 and pertaining to group 1 is 1.00.  Table 11 shows the confusion matrix.

As shown in Tables 12 and 13, our proposed model was performed on two German stock indices, likely AFX.DE and MRK.DE, whose performances and accuracy measures are shown in the table. From the recorded results, we found that the CVS.F index has a training accuracy of 99.1627, while the testing accuracy is 99.1631. The recorded AUC score is 99.15, and Hamming loss is 0.00836. The precision value recorded as group 0 is 0.99 and group 1 is 1.00. In contrast, the recall value recorded related to group 0 is 1.00 and about group 1 is 0.99, and the *f*1 score related to group 0 is 99.00 and related to group 1 is 99.00. For the index MRK.DE, the training accuracy is 99.4553, while the testing accuracy is 99.4771. The recorded AUC score is 99.15, and the Hamming loss is 0.00522. The precision value recorded in group 0 is 0.99 and in group 1 is 1.00. In contrast, the recall value recorded related to group 0 is 1.00 and related to group 1 is 0.99, and finally, the *f*1 score about group 0 is 99.00 and related to group 1 is 99.00.  Table 11 shows the confusion matrix.

As shown in Table 14, our proposed model was performed on one London stock index, likely DPH.L, whose performances and accuracy measures are shown in Table 14. From the recorded results, we found that the DPH.L index has a training accuracy of 98.74, whereas the testing accuracy is 98.40. The recorded AUC score is 98.44, and the Hamming loss is 0.01591. The precision value recorded pertaining to group 0 is 0.97 and pertaining to group 1 is 1.00. In contrast, the recall value recorded pertaining to group 0 is 1.00 and pertaining to group 1 is 0.97, and finally, the f1 score pertaining to group 0 is 98.00 and about group 1 is 98 .00. The forecast performances of online learning ensemble model, London indices.  Table 15 shows the confusion matrix.

### 5.1. Performance Comparison with Past Works

Table 16 shows a relative performance level with the past works with our proposed model.

### 5.2. Practical Implications

Nowadays, machine learning-based systems give recommendations about certain companies to investors to have a basic notion and minimize their investing losses. By analysing vast quantities of data and developing simple, widely accessible solutions that benefit everyone, not just businesses, AI has a big impact on the exchange of currency. In contrast to humans, who appear to be overly enthusiastic about the trading of assets, AI will make reasoned, accurate, and fair speculative decisions. This strategy could be used to develop new trading strategies or to manage investment portfolios by rebalancing holdings based on trend forecasts. This will help various financial institutions collect information about share prices so they can advise their clients on how to maximize earnings and reduce losses. In addition, it pushes the research community on a new path by illustrating how online algorithms can be combined with various technical markers and the ramifications of modifying various parameters [102, 113].

## 6. Conclusion

In this manuscript, we come up with a method that uses an ensemble-based incremental learning approach for short-term stock price forecasting that can minimize investment risk (an optimal portfolio) and make a predictive decision to find the direction of the selected stocks. Our proposed framework is composed of two models. The first model is the mean-variance model, which is used to minimize the risk assessment of individual stocks. The second model is an incremental-based ensemble model composed of two online learning algorithms, i.e., perceptron and passive-aggressive classifier. Besides the historical data of stock market closing prices, open price volume, nine indicators, and two extracted features are also inserted to boost the ensemble framework's performance. Initially, we selected 20 stocks from four different geographic countries, i.e., America, Germany, France, and London. Still, after implementing the mean-variance model, we got only 10 indices for the final experiment. Our fact-finding technique revealed that, as the stock market is always a source of data collection at regular intervals, it is always a good perspective from a researcher's point of view. Therefore, instead of using a batch learning technique, an online learning technique is used for forecasting the financial market. Our experimental bench reveals that the random selection of stock market data for forecasting is a meaningless practice for the researcher, which should be avoided with the help of different portfolio selection techniques. As a reference, our proposed framework revealed a lot. Our study found that our proposed online learning ensemble models' performance, which is depicted in Table 17, shows the average accuracy level of indices belonging to four different geographic reasons.

The study revealed that performance levels may increase when we add indicators as our input features and handle the imbalanced dataset. Therefore, instead of using a batch learning-based single predictive model for forecasting, it is always better to practice using an ensemble model based on online learning to improve forecasting performances, as shown in Table 16. However, if we look into the runtime of this framework, it takes the range of training time, i.e., 4 to 12 seconds to train the model on different datasets. As the number of folds increases, the training time also increases, as well as when the data size increases, the training time also increases.

### 6.1. Limitations and Future Work

However, despite the excellent prediction performance of our proposed methodology, certain limitations may be resolved in the future. First, as our current study only predicts the direction of stocks one day in advance, it will require growth in the future for long-term market direction predictions. This study was limited to four stock exchanges, but it could have been more comprehensive if it had included stock exchanges from additional countries. In conclusion, future research must also employ this dataset, as the study did not investigate additional information sources, such as fundamental and sentiment analysis [52, 105, 106, 112–114].

## Figures and Tables

**Algorithm 1:**
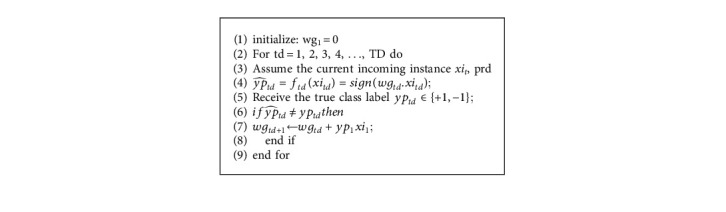
Algorithm 1 Perceptron.

**Table 1:** Numerous research studies conducted based on batch learning techniques.

SL. No.	Authors (year)/publisher	Dataset used	Target output	Period of forecasting	Preferred technique
1	Jiang et al. (2019) [98]	Three major US stock indices (S&P 500, Dow 30, Nasdaq)	Market direction	Short	Tree-based ensemble method + deep learning
2	Ayala et al. (2021) [99]	IBEX, DAX, and DJIA	Stock index prediction	Short span (for a particular window)	Linear regression and ANN regression model performed well among all ML models
3	Nabipour et al. (2020) [100]	Tehran Stock Exchange	Price prediction	Short term	Technical indicators + LSTM
4	Shafiq et al. (2020) [101, 102]	Chinese stock market	Index trend	Short-time period	Feature engineering-based fusion model using PCA and LSTM
5	Jothimani and Yadav (2019) [28]	Nifty Index	Asset price	Short span	CEEMDAN, ANN, SVR, EEMD, EMD
6	Zhong and Enke (2019) [44]	US SPDR S&P 500 ETF (SPY)	Daily return direction	Short term	Fusion of deep neural network and principal component analysis.
7	Ampomah et al. (2020) [50]	NYSE, Nasdaq, NSE	Daily return direction	Short term	A comparative study done using different tree-based ensemble models where extra tree performs better
8	Sun et al. (2020) [103]	Chinese stock market	Return direction of asset	Short period	AdaBoost-SVM + SMOTE
9	Yang et al. (2020) [104]	Shanghai and Shenzhen 300 Index	Market volatility forecast	Short term	SVM
10	Jiayu et al. (2020) [62]	S&P 500, DJIA, HSI	Index price	Short term	Long short memory with attention mechanism
11	Boonpeng and Jeatrakul (2016) [57]	Thailand Stock Exchange	Index price	Short term	OAA-neural network
12	Yang et al. (2019) [58]	China Stock Exchange	Market volatility	Intra-day	SVM
13	Yun et al.(2021) [68]	Apple and Yahoo	Index price	Short term	XGBoost
14	Ecer et al. (2020) [63]	Borsa Istanbul	Return direction of asset	Short term	MLP–GA, MLP–PSO
15	Wang et al. (2012) [70]	Shenzhen Integrated Index and DJIA	Index price	Weekly	BPNN, ARIMA, and ESM
16	Chenglin et al., (2020) [71]	China Stock Exchange	Trend of stock market	Short term	ARI-MA-LS-SVM
17	Tiwari et al. (2010) [72]	BSE, India	Index price	Short term	Markov model + decision tree
18	Paiva et al.(2018) [85]	IBOVESPA	Portfolio selection and stock prediction	Dailly	SVM+ mean-variance
19	Wang et al. (2020) [86]	US Stock Exchange 100 Index	Portfolio selection and stock prediction	Return per year	LSTM + mean-variance
20	Basak et al. (2018) [25]	10 Indian Stock Exchange Companies	Index price increase or decrease	Medium to long run	XGBoost + random forest

**Table 2:** List of stocks initially selected for an experiment with different geographic areas.

USA	London	Germany	France
DHR	AZN	AFX	CGN
JNJ	DPHL	BAYN	CVS
MDT	GSK	BRM	EWL
NVO	HIKL	MRK	MTO
UNH	SNL	SRT	TN8

**Table 3:** List of different geographic stocks finally selected for experiment.

Name of the stocks	DHR	NVO	UNH	DPHL	AFX	MRK	CVS	EWL	MTO	TN8
Range of the dataset	5/11/1987 to 24/11/2021	04/01/1982 to 24/11/2021	26/03/1990 to 24/11/2021	21/09/2000 to 24/11/2021	22/03/2000 to 24/11/2021	26/06/1998 to 24/11/2021	03/01/2000 to 24/11/2021	08/03/2001 to 24/11/2021	11/09/2000 to 24/11/2021	11/09/2000 to 24/11/2021

DHR- Danaher Corporation; NVO- Novo Nordisk A/S; UNH- UnitedHealth Group Incorporated; DPHL- Dechra Pharmaceuticals PLC; AFX- Alpha FX Group plc; MRK -Merck & Co., Inc.; CVS - CVS Health Corporation; EWL - iShares; MSCI Switzerland ETF; MTO -Mitie Group plc; TN8 - Thermo Fisher Scientific Inc.

**Table 4:** List of American stocks with their weights.

Name of the stocks	DHR	JNJ	MDT	NVO	UNH
Weights	0.04507	**0.0**	**0.0**	0.69592	0.25901

DHR- Danaher Corporation; JNJ- Johnson & Johnson; MDT- Medtronic plc; NVO- Novo Nordisk A/S; UNH- UnitedHealth Group Incorporated.

**Table 5:** List of United Kingdom stocks with their weights.

Name of the stocks	AZN	DPHL	GSK	HIKL	SNL
Weights	0.0	**1.0**	**0.0**	0.0	0.0

AZN- AstraZeneca PLC; DPHL- Dechra Pharmaceuticals PLC; GSK- GlaxoSmithKline plc; HIKL- Hikma Pharmaceuticals PLC; SNL - smith & nephew plc.

**Table 6:** List of Germany stocks with their weights.

Name of the stocks	AFX	BAYN	BRM	MRK	SRT
Weights	0.31381	0.0	0.0	0.68619	0.0

Annual volatility: 20.7%; Sharpe ratio: 2.80.

**Table 7:** List of France stocks with their weights.

Name of the stocks	CGN	CVS	EWL	MTO	TN8
Weights	0.0	**0.23574**	**0.36912**	0.16845	0.22668

Annual volatility: 15.6%; Sharpe ratio: 2.37.

**Table 8:** Forecast performances of the online learning ensemble model, American indices.

	Accuracy		AUC score	Hamming loss	Precision		Recall		*f*1 score	
	Train	Test			0	1	0	1	0	1
UNH	99.16	99.60	99.6041	0.00399	0.99	1.00	1.00	0.99	1.00	1.00
NVO	99.68	99.73	99.7322	0.00268	0.99	1.00	1.00	0.99	1.00	1.00
DHR	99.47	99.36	99.3724	0.00635	0.99	1.00	1.00	0.99	99.00	99.00

**Table 9:** Confusion matrix of American indices.

	TP	FP	TN	FN
UNH	1013	2	984	6
NVO	1300	0	1302	7
DHR	1105	1	1083	13

**Table 10:** Forecast performances of the online learning ensemble model, France indices.

	Accuracy		AUC score	Hamming loss	Precision		Recall		*f*1 score	
	Train	Test			0	1	0	1	0	1
CVS.F	99.72	99.49	99.50	0.00508	99.00	1.00	1.00	99.00	99.00	1.00
EWL.F	99.87	99.61	99.60	0.00386	99.00	1.00	1.00	99.00	1.00	1.00
MTO.F	99.94	99.91	99.91	0.00086	1.00	1.00	1.00	1.00	1.00	1.00
TN8.F	99.81	99.84	99.84	0.00151	1.00	1.00	1.00	1.00	1.00	1.00

**Table 11:** Confusion matrix of France indices.

	TP	FP	TN	FN
CVS.F	998	0	958	10
EWL.F	11013	1	1049	7
MTO.F	1162	0	1151	2
TN8.F	989	0	984	3

**Table 12:** Forecast performances of the online learning ensemble model, German indices.

	Accuracy		AUC score	Hamming loss	Precision		Recall		*f*1 score	
	Train	Test			0	1	0	1	0	1
AFX.DE	99.1627	99.1631	99.15	0.00836	99.00	1.00	1.00	99.00	99.00	99.00
MRK.DE	99.4553	99.4771	99.47	0.00522	99.00	1.00	1.00	99.00	99.00	99.00

**Table 13:** Confusion matrix of German indices.

	TP	FP	TN	FN
AFX.DE	702	2	720	10
MRK.DE	762	1	760	7

**Table 14:** Forecast performances of the online learning ensemble model, London indices.

	Accuracy		AUC score	Hamming loss	Precision		Recall		f1 score	
	Train	Test			0	1	0	1	0	1
DPH.L	98.74	98.40	98.44	0.01591	97.00	1.00	1.00	97.00	98.00	98.00

**Table 15:** Confusion matrix of London indices.

	TP	FP	TN	FN
DPH.L	748	0	736	24

**Table 16:** Comparison of historical stock market forecasting methods to our suggested model's performance.

Author	Output	Performance measurement (accuracy) (%)
Malagrino et al. (2018) [109]	Daily asset flow	71–78
Zho et al. (2018) [110]	Daily asset flow	66.67
Zheng et al. (2018) [107, 111]	Daily asset flow	79.4
Ren et al. (2018) [112]	Daily asset flow	89.0
Hu et al. (2018) [113]	Daily asset flow	89.0
Fischer and Krauss (2018) [114]	Daily asset flow	56
Our proposed model	Daily asset flow	**98-99**

**Table 17:** Average accuracy of the proposed framework of different geographic indices.

Country	America	German	France	London
Average accuracy	99.56	99.32	99.71	98.40

## Data Availability

The data used to support the findings of this study are available from the first author upon request.
